# miR-203 inhibition of renal cancer cell proliferation, migration and invasion by targeting of FGF2

**DOI:** 10.1186/s13000-015-0255-7

**Published:** 2015-04-09

**Authors:** Mingxi Xu, Meng Gu, Ke Zhang, Jun Zhou, Zhong Wang, Jun Da

**Affiliations:** Department of Urology, Shanghai Ninth People’s Hospital, Shanghai Jiaotong University, Shanghai, 200011 China

**Keywords:** Renal cancer, miR-203, FGF2, Progression

## Abstract

**Background:**

Renal cell carcinoma (RCC) is one of the leading causes of cancer related mortality worldwide. Increasing evidence has shown that microRNAs function as oncogenes or tumor suppressors in human malignancies, but the roles of miR-203 in human RCC is still unclear.

**Methods:**

First, quantitative real-time PCR (qRT-PCR) was performed to detect miR-203 expression in renal cancer cell lines and clear cell RCC (ccRCC) specimens. Then, the association of miR-203 expression with clinicopathological features and survival was later analyzed. Finally, the roles of miR-203 in regulation of tumor proliferation, migration, invasion, and target gene expression were further investigated.

**Results:**

Our study showed miR-203 was down-regulated in renal cancer cell lines and ccRCC specimens (P < 0.05). Respectively, the low miR-203 expression in ccRCC specimens was associated with advanced clinical features and poorer prognosis (P < 0.05). miR-203 expression was an independent prognostic marker of overall ccRCC patient survival in a multivariate analysis (P < 0.05). Transient forced expression of miR-203 inhibited renal cancer cell growth and metastasis (P < 0.05). In contrast, down-regulation of miR-203 expression promoted renal cancer cell growth and metastasis (P < 0.05). Mechanistic investigations confirmed FGF2 as a direct target of miR-203, and up-regulation of miR-203 could decrease expression of FGF2. Further investigation showed that ectopic expression of FGF2 partially reversed the inhibition effect of enforced miR-203 expression on the malignant phenotypes of renal cancer cells.

**Conclusions:**

Our study suggested that miR-203 could be a potential prognostic marker and functions as a tumor suppressor in human renal cancer by post-transcriptionally targeting FGF2.

**Virtual slides:**

The virtual slide(s) for this article can be found here: http://www.diagnosticpathology.diagnomx.eu/vs/6828145701534108.

## Background

Renal cell carcinoma (RCC) is the most common solid cancer of the adult kidney, accounting for approximately 90% of kidney neoplasms and 3% of all adult malignancies [1]. Worldwide mortality, as a result of RCC, currently exceeds 100,000 patients each year, with the incidence and mortality rate increasing by 2–3% per-decade [2]. Although radical nephrectomy is effective to cure early and local RCC, 20–40% of patients develop metastatic disease after surgery [3]. Patients with metastatic RCC face a dismal prognosis and have limited therapeutic options. Median survival in a recent cohort was only 1.5 years with fewer than 10% of patients surviving to 5 years [4]. Therefore, studying the molecular basis of RCC is crucial for designing new therapeutic agents that will improve the survival rate.

MicroRNAs (miRNAs) are small, single stranded, non-protein coding RNAs of 22 nucleotides that are capable of silencing gene expression at the post-transcriptional level [5]. Computational predictions of miRNA targets suggest that miRNAs may regulate more than 30% of human protein-coding genes [6]. Since miRNAs were first reported to show anticancer effects in patients with B cell chronic lymphocytic leukaemia, these molecules have been shown to be critical in carcinogenesis [7]. Moreover, alterations in miRNA expression have been proven to play key roles in a wide range of physiologic and pathologic processes, including cell proliferation, migration, apoptosis, development, and metabolism [8-10]. Recent studies showed that several miRNAs have been implicated in the development and progression of renal cell carcinoma, such as miR-646, miR-21 and miR-204 [11-13]. However, miRNAs and their roles in renal cell carcinoma remain largely unknown.

MiR-203 located at chromosome 14q32-33 and has been identified as a skin-specific keratinocyte derived miRNA involved in keratinocyte differentiation [14]. Tian et al. showed miR-203 expression was significantly lower in laryngeal squamous cell carcinoma and correlated with poor differentiation, advanced clinical stages and lymph node metastasis [15]. Diao et al. revealed that miR-203 exerted its tumor suppressive effect by directly targeting p63 and leukemia inhibitory factor receptor in rhabdomyosarcoma cells, which promoted myogenic differentiation by inhibiting the Notch and the JAK1/STAT1/STAT3 pathways [16]. Wang et al. demonstrated that miR-203 suppressed the proliferation and migration and promoted the apoptosis of lung cancer cells by targeting SRC [17]. Zhang suggested that miR-203 suppressed tumor growth and invasion through repressing Ran in esophageal cancer [18]. Siu et al. found that loss of EGFR signaling-regulated miR-203 promotes prostate cancer bone metastasis and tyrosine kinase inhibitors resistance [19]. However, the dysregulation of miR-203 and its possible involvement in renal cell carcinoma has not been reported.

In this study, first, we investigated the expression of miR-203 in renal cancer cells and ccRCC tissues. Then, the association of miR-203 expression with clinicopathologic features of ccRCC patients and its prognostic value was explored. Finally, experiments in vitro further confirmed that forced miR-203 expression could inhibit cell proliferation, migration and invasion of renal cancer cells by down-regulating FGF2 expression, which further elucidates the molecular mechanism involved in renal cancer progression and may suggest novel findings for targeted treatment.

## Methods

### Human ccRCC specimens

A total of 90 primary ccRCC tissues and matched adjacent non-tumor tissues were obtained from patients who underwent radical nephrectomy in the Department of Urology, Shanghai Ninth People’s Hospital between 2005 to 2008. The 90 patients included 50 men and 40 women with a median age of 57.3 years (range from 43 to 79 years). The follow-up time for these patients ranged from 2 to 60 months with a median follow-up time of 35 months. None of the patients had received chemotherapy or radiotherapy before surgery. After surgical resection, tumor specimens and adjacent non-tumor tissues were collected and stored in liquid nitrogen until use. Clinicopathological features in our study were shown in Table 1. Clinical sample cohorts used for this study were approved by the Institution research ethics committee of Shanghai Jiaotong University. All participants gave written consent of their information to be stored in the hospital database and used for research.

**Table 1:** **The relationship between miR-203 expression and clinicopathologic features in ccRCC**

**Parameters**	**Group**	**Total**	**miR-203 expression**		**P value**
			**High**	**Low**	
Gender	Male	50	19	31	0.665
	Female	40	17	23	
Age (years)	<65	49	20	29	0.863
	≥65	41	16	25	
Tumor size (cm)	<4 cm	57	26	31	0.153
	≥4 cm	33	10	23	
Histological grade	G1-G2	59	31	28	0.001
	G3-G4	31	5	26	
Tumor stage	T1-T2	54	28	26	0.005
	T3-T4	36	8	28	
Lymph nodes metastasis	Absence	74	34	40	0.013
	Presence	16	2	14	

### Cell culture and cell transfection

Immortalized normal human proximal tubule epithelial cell line HK-2 was purchased from the American Type Culture Collection (ATCC, USA). Human renal cancer cell lines 786-O, ACHN, Caki-1, and Caki-2 were obtained from the Cell Bank of Type Culture Collection of Chinese Academy of Sciences (CCCAS, China). HK-2 cells were cultured in KSFM medium (Gibco), and other cells were cultured in RPMI-1640 medium (Gibco) with 10% fetal bovine serum (FBS, Gibco), 50 U/ml of penicillin and 50 μg/ml of streptomycin. All cells were cultured in a sterile incubator maintained at 37°C with 5% CO_2_.

786-O cells were seeded in 12-well plates and incubated overnight, then transiently transfected with miR-203 mimic, miR-negative control of mimics (miR-Ctrl), miR-203 inhibitor and miR-negative control of inhibitor (anti-miR-Ctrl) using Lipofectamine 2000 (Invitrogen) according to the manufacturer’s instructions. MiR-203 mimic and miR-203 inhibitor were obtained from Applied Biosystems. The full length FGF2 cDNA (which included the ORF and 3’UTR) was PCR-amplified and cloned into the pcDNA3.1 vector to generate the pcDNA-FGF2 constructs that were used in the rescue assays. 786-O cells in 12-well plates were co-transfected with miR-203 mimic and the pcDNA-FGF2 plasmid.

### Cell viability assay

The viability of 786-O cells was determined using the Cell Counting Kit-8 (CCK-8, Dojindo) according to the manufacturer’s instructions. Briefly, 786-O cells were plated at 5 × 10^3^ cells per well in 96-well plates and incubated overnight in RPMI-1640 medium supplemented with 10% FBS. After transfection, 10 μl CCK-8 liquid was added to the test well and incubated for 3 h. Absorbance was then measured at a wavelength of 450 nm.

### Transwell migration and invasion assays

The migration and invasion assays were carried out using Transwell insert chambers (Corning). For migration assay, 1x10^4^ cells were plated into the upper chamber in serum-free medium in triplicate. Medium containing 10% FBS in the lower chamber served as chemoattractant. After incubation for 24 h, cells in the upper chambers were removed by wiping with a cotton swab and cells migrated to the lower surface of filter were fixed in 70% ethanol for 30 min and stained with 0.2% crystal violet for 10 min. Cell migration was scored by counting five random fields per filter under a light microscope. For invasion assay, 2x10^4^ cells were seeded into upper chambers precoated with matrigel (BD) in serum-free medium in triplicate. Medium with 10% FBS was added to the lower chamber to serve as chemoattractant. After incubation for 48 h, non-invading cells on the upper surface of filter were removed with cotton swabs and invading cells that migrated to the lower surface of filter were fixed, stained and scored as described above.

### miRNA target prediction

The miRNAs that may target FGF2 were determined using algorithms from TargetScan (http://genes.mit.edu/targetscan/), PicTar (http://pictar.mdc-berlin.de/), and miRanda (http://cbio.mskcc.org/cgi-bin/mirnaviewer/mirnaviewer.pl).

### Luciferase reporter assay

The 3′ untranslated region (UTR) of human FGF2 gene that was predicted to interact with miR-203 was synthesized and inserted into pMIR-REPORT (Ambion), yielding pMIR-REPORT FGF2. Mutations within potential miR-203 binding sites were generated by nucleotide replacement of wild-type sequence to inhibit miR-203 binding. Cells were transfected with the pMIR-REPORT vectors containing the 3′UTR variants and miR-203 mimics for 24 h. Luciferase values were determined using the Dual-Luciferase Reporter Assay System (Promega).

### Total RNA extraction and quantitative real-time PCR

Total RNAs were isolated using TRIzol (Invitrogen) according to the manufacturer’s protocol. For mRNA quantitative assay, total RNAs were reverse transcribed using the reverse transcription kit (Promega) according to the manufacturer’s guidelines. qRT-PCR was performed using the SYBR Green Master Mix (Takara). GAPDH was used as the internal control. For miRNA quantitative assay, TaqMan assays (Applied Biosystems) were performed according to the manufacturer’s instructions. The U6 snRNA and GAPDH were used as endogenous control for miRNA and mRNA, respectively. The ΔΔCt method was used to determine relative quantitation of miRNA and mRNA expression, and fold change was determined as 2^-ΔΔCt^.

### Western blot

Cultured cells were lysed in lysis buffer containing protease inhibitor. Protein concentration was determined using a Bio-Rad protein assay system (Bio-Rad). Equivalent amounts of proteins were separated by SDS-PAGE, and then transferred to polyvinylidene difluoride membranes (Bio-Rad). After being blocked in Tris buffered saline (TBS) containing 5% nonfat milk, the membranes were incubated with specific primary antibodies (Abcam) at 4°C for 12 hours and then with horseradish peroxidase conjugated anti-mouse antibody for 2 hours at room temperature. ECL detection reagent (Amersham LifeScience) was used to demonstrate the results.

### Statistical analysis

All statistical analyses were performed using SPSS version 18.0 software (IBM). The significance of differences between groups was estimated by Student’s t-test, χ2 test or Wilcoxon test, as appropriate. Overall survival rates were calculated by the Kaplan-Meier method with the log-rank test applied for comparison. Survival data were evaluated using univariate and multivariate Cox proportional hazards model. Variables with a value of p < 0.05 in univariate analysis were used in subsequent multivariate analysis on the basis of Cox regression analyses. Correlation between expression levels of miR-203 and its target genes in RCC tissues was analyzed using Spearman’s correlation coefficient. Two-sided p-values were calculated, and a probability level of 0.05 was chosen for statistical significance.

## Results

### miR-203 expression was down-regulated in renal cancer and negatively correlated with advanced clinical stage

**Figure 1:**
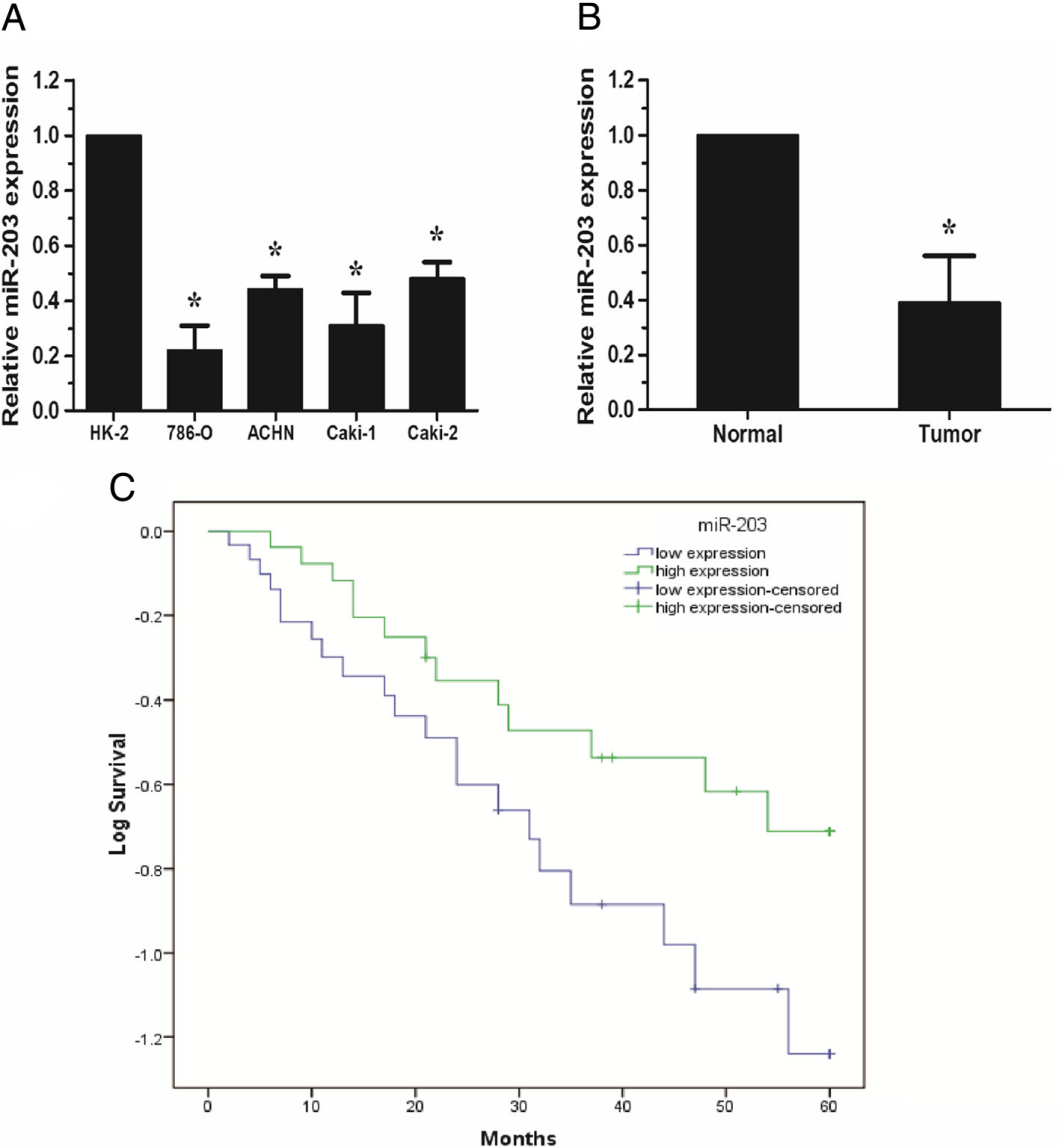
**Low expression of miR-203 was found in ccRCC tissues and associated with poorer overall survival. A**. The expression level of miR-203 was lower in renal caner cell lines compared with normal human proximal tubule epithelial cell line HK-2. **B**. The expression levels of miR-203 were significantly decreased in ccRCC tissues compared to adjacent non-tumor tissues. **C**. The Kaplan–Meier analysis revealed that low expression of miR-203 was associated with poorer overall survival of ccRCC patients.* P <0.05.

qRT-PCR was performed to detect the expression of miR-203 in human renal cancer cell lines (786-O, ACHN, Caki-1 and Caki-2) and normal human proximal tubule epithelial cell line HK-2. We found that the average expression of miR-203 was down-regulated in 4 renal cancer cell lines when compared with the normal human proximal tubule epithelial cell line HK-2 (Figure 1A, P < 0.05). To explore whether miR-203 was down-regulated in renal cancer tissues, the expression of miR-203 was determined in 90 pairs of ccRCC tissues and adjacent non-tumor tissues. The expression of miR-203 in ccRCC tissues was significantly down-regulated (Figure 1B, P < 0.05).

Then, patients with ccRCC were classified into two groups based on the mean value (0.38) of relative miR-203 expression. The high miR-203 group had miR-203 expression levels higher than the mean value and the low miR-203 group had miR-203 expression levels lower than the mean value. As showed in Table 1, miR-203 expression was significantly associated with histological grade, tumor stage and lymph node metastasis (Table 1, P < 0.05), but no significant association with patients’ gender, age and tumor size (Table 1, P > 0.05). Taken together, these results indicated that miR-203 was down-regulated in renal cancer, and a reduced expression of miR-203 may play key roles in the progression of renal cancer.

### Low miR-203 expression predicted poor prognosis in patients with RCC

Kaplan-Meier survival analysis showed that patients with low miR-203 expression (31.2, 95% CI: 23.5-38.9) had shorter overall survival than those with high miR-203 expression (42.2, 95% CI: 34.3-49.9) (Figure 1C, P < 0.05, log-rank test). As in Table 2, Univariate analysis showed that the overall survival of patients with ccRCC was associated with miR-203 expression level, histological grade, tumor stage and lymph node metastasis (P < 0.05). Furthermore, multivariate analysis showed that relative expression of miR-203, histological grade, tumor stage and lymph node metastasis were independent prognostic factors for overall survival of ccRCC patients (Table 2). These results suggested that miR-203 expression level can be developed as a powerful independent molecular biomarker for the predicting of overall survival (HR: 3.071, 95% CI: 1.719-6.374, P = 0.001) in ccRCC patients.

**Table 2:** **Univariate and multivariate analyses of prognostic parameters for survival in patients with ccRCC**

**Variable**	**Univariate analysis**			**Multivariate analysis**		
	**HR**	**95% CI**	***P***	**HR**	**95% CI**	***P***
Gender	1.134	0.738-1.927	0.214			
Male vs Female						
Age (years)	1.442	0.672-2.154	0.195			
≥65 vs <65						
Tumor size	1.827	0.731-3.742	0.206			
≥4 vs <4						
Histological grade	2.917	1.362-5.514	0.002	2.722	1.291-5.342	0.007
G3-G4 vs G1-G2						
Tumor stage	2.724	1.574-6.291	0.012	2.572	1.427-6.073	0.018
T3-T4 vs T1-T2						
Lymph node	3.721	2.142-7.692	0.008	3.461	1.975-6.824	0.013
Presence vs Absence						
miR-203	3.327	1.628-6.927	0.007	3.071	1.719-6.374	0.001
Low vs High						

### miR-203 inhibited cell proliferation, migration and invasion of renal cancer cells

**Figure 2:**
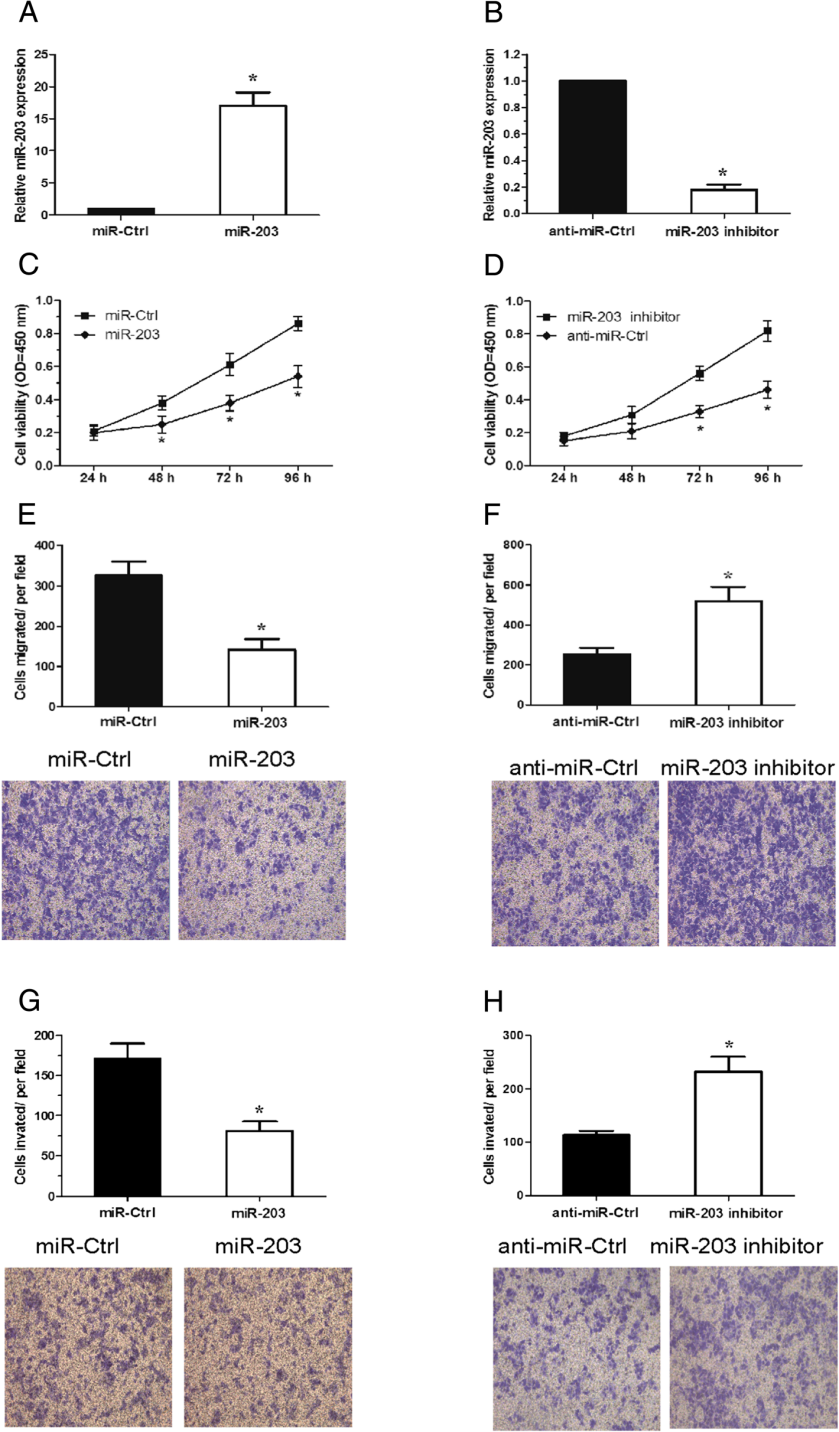
**Effects of miR-203 expression on proliferation, migration and invasion of renal cancer cells. A**. Over-expression of miR-203 by transfection with miR-203 mimics in 786-O cells. **B**. Down-regulation of miR-203 by transfection with miR-203 inhibitor in 786-O cells. **C,D**. CCK-8 assay was performed to analyze the effect of miR-203 on cell proliferation of 786-O cells. **E-H**. Transwell migration and invasion assays were utilized to analyze the effect of miR-203 on cell migration**(E,F)** and invasion**(G,H)** of 786-O cells.* P <0.05.

To characterize the function of miR-203 in renal cancer, 786-O cells were transiently transfected with miR-203 mimics or miR-203 inhibitor, and the expression of miR-203 was determined by qRT-PCR (P < 0.05, Figure 2A, B). CCK-8 assay was used to examine the proliferation of renal cancer cells, our data showed that the proliferation rate of 786-O cells transfected with miR-203 mimics was clearly decreased (P < 0.05, Figure 2C). In contrast, the proliferation rate of 786-O cells transfected with miR-203 inhibitor was increased (P < 0.05, Figure 2D). Similarly, in vitro migration and invasion assays showed that over-expression of miR-203 inhibited the migration and invasion ability of 786-O cells (P < 0.05, Figure 2E, G). Consistent with this result, silencing of miR-203 resulted in a significant increase in renal cancer cell migration and invasion ability (P < 0.05, Figure 2F, H). These results demonstrated that miR-203 was able to suppress the progression of renal cancer.

### miR-203 directly target FGF2 in human renal cancer cells

**Figure 3:**
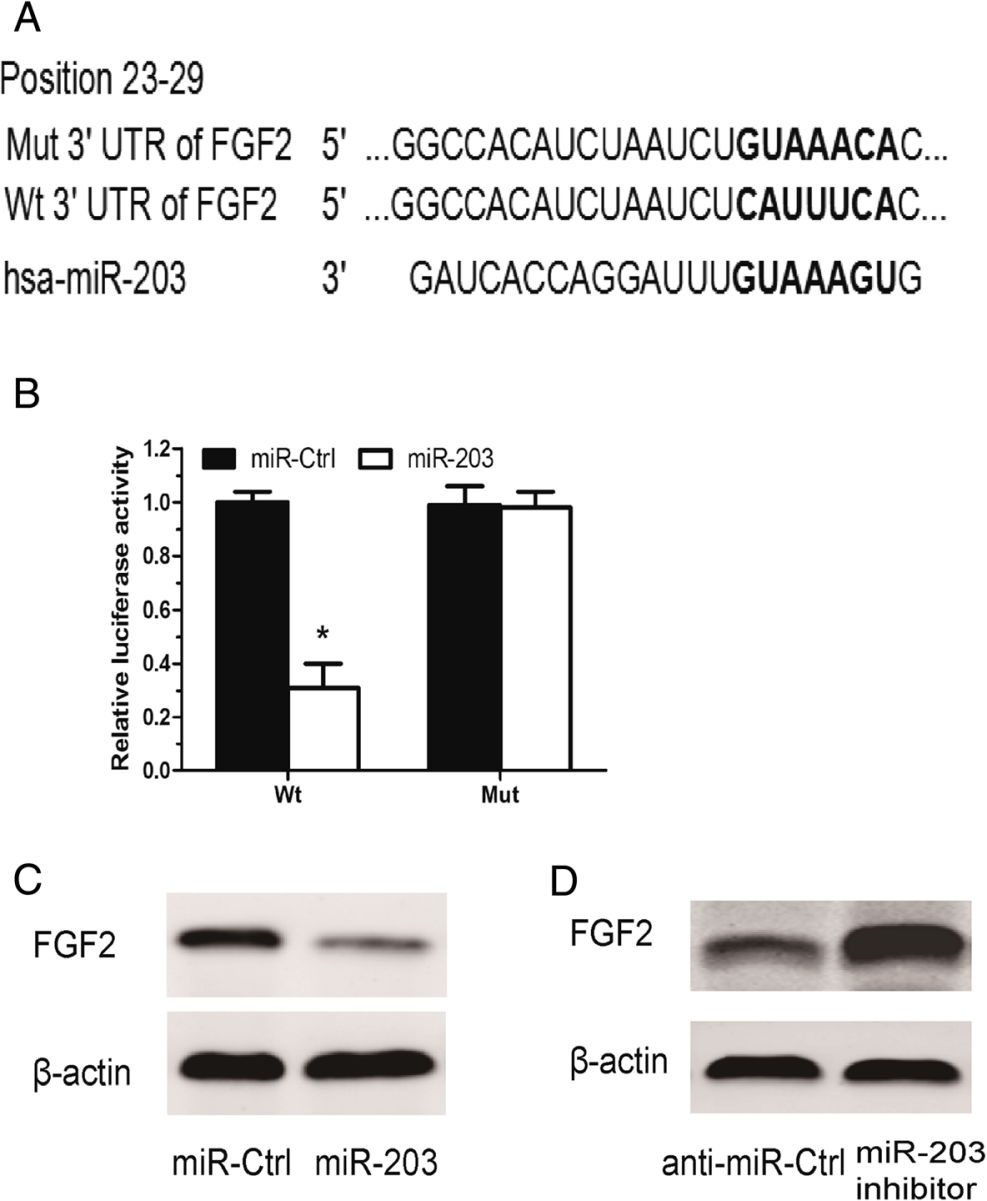
**miR-203 directly targets FGF2 in renal cancer cells. A**. The wild type and mutant complementary sequences of the FGF2 mRNA 3’UTR are shown with the miR-203 sequence. **B**. Luciferase assay in 786-O cells co-transfected with miR-203 mimic and a luciferase reporter containing the FGF2 3’UTR (Wt) or a mutant (Mut). **C**. Overexpression of miR-203 inhibited the expression of FGF2 at the protein level in 786-O cells. **D**. Down-regulated expression of miR-203 increased the expression of FGF2 at the protein level in 786-O cells. * P <0.05.

Based on miR target analysis using the websites targetscan, PicTar and miRanda, miR-203 was identified as a potential regulator of FGF2 expression. The predicted binding of miR-203 with FGF2 3’UTR was shown (Figure 3A), suggesting that miR-203 might be a potential miRNA-targeting FGF2. To confirm that FGF2 is the direct target of miR-203, luciferase reporter assay was performed. Our result showed that miR-203 significantly inhibited the luciferase activity of the wild-type (Wt) 3′UTR of FGF2, without effect on its mutant (Mut) (P < 0.05, Figure 3B). Moreover, western blot analysis was used to detect the expression of FGF2 regulated by miR-203 in 786-O. Our results showed that miR-203 mimics significantly inhibited expression of FGF2 (Figure 3C). Whereas silencing of miR-203 significantly increased expression of FGF2 (Figure 3D). These data suggested that FGF2 was a target of miR-203 in renal cancer cells.

### FGF2 over-expression partially attenuated the tumor suppressive effect of miR-203

**Figure 4:**
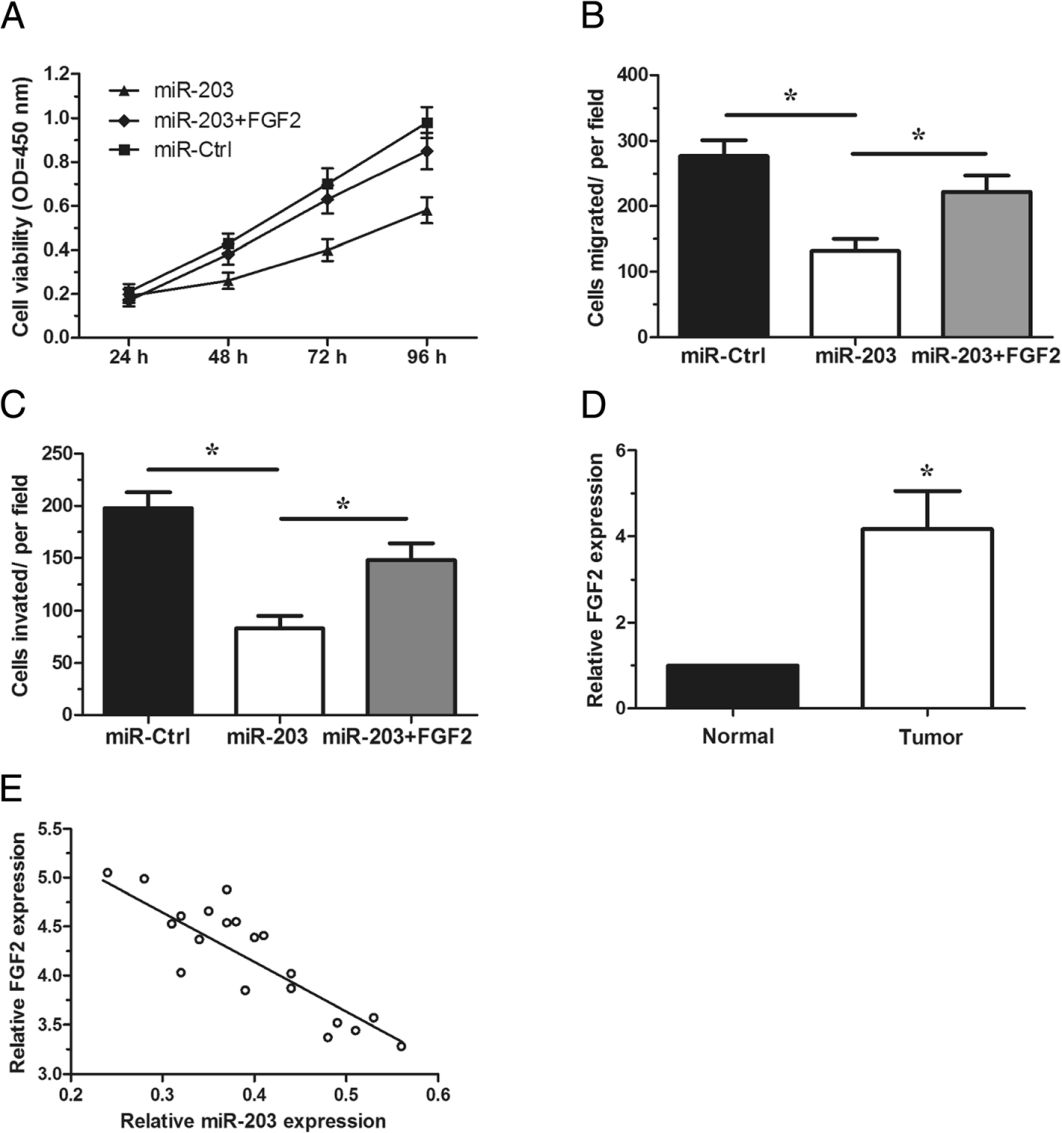
**FGF2 over-expression partially attenuated the tumor suppressive effect of miR-203. A–C**. 786-O cells were transfected with miR-203 mimics or miR-Ctrl with FGF2 over-expression plasmid. CCK-8 assay **(A)**, in vitro migration assay **(B)** and in vitro invasion assay **(C)** were performed. **D**. The relative expression levels of FGF2 in 20 ccRCC tissues and adjacent non-tumor tissues were determined. **E**. The inverse correlation between FGF2 and miR-203 expression in 20 ccRCC samples was determined using Spearman’s correlation analysis (r = −0.8340, P <0.05) * P <0.05.

We further investigated whether FGF2 over-expression could attenuate the tumor suppressive effects of miR-203. CCK-8 assay, migration assay and invasion assay revealed that over-expression of FGF2 significantly reversed the tumor suppressive effects of miR-203 on 786-O cell (P < 0.05, Figure 4A-C). Taken together, these findings indicated that restoration of FGF2 markedly attenuated the tumor suppressive effect of miR-203.

### FGF2 was up-regulated and negatively correlated with miR-203 expression in ccRCC

To further explore the relationship between FGF2 and miR-203 in vivo, qRT-PCR was used to examine the expression of FGF2 in one set of ccRCC tissue and their matched non-tumor tissues (20 pairs). We found that FGF2 expression was significantly increased in ccRCC tissues relative to the matched non-tumor tissues (P < 0.05, Figure 4D). Moreover, our data indicated that there was an inverse correlation between miR-203 and FGF2 expression (P < 0.05, Figure 4E). Our data further demonstrated that FGF2 was a target of miR-203.

## Discussion

Despite the advancements in treatment options, improvements in renal cancer patient survival have been limited owing to lack of early detection [20]. Biomarkers to improve renal cancer diagnosis, prognosis and prediction of treatment response therefore represent opportunities to improve patient outcome [21]. In recent years, investigation of epigenetic biomarkers such as miRNA expression has implicated that these alterations may be enticing translational biomarker candidates in renal cancer.

In the present study, our data showed that the expression of miR-203 was significantly down-regulated in renal cancer cells and ccRCC tissues compared with normal human proximal tubule epithelial cells and adjacent non-tumor tissues. Decreased expression of miR-203 was associated with a more aggressive tumor phenotype in ccRCC patients. In addition, overall survival of patients with low miR-203 expression was less than those with high expression of miR-203. From those data, we concluded that decreased expression of miR-203 would play a critical role in the progression of renal cancer.

The fibroblast growth factor (FGF) family comprises at least 23 members, which function through four-transmembrane tyrosine kinase receptors on the surface of target cells [22]. Among the FGF family members, fibroblast growth factor-2 (FGF2) is well characterised, bearing all typical features of the FGF family, and is regarded as a prototypic growth factor [23]. Recent studies indicated that FGF2 was an important proangiogenic growth factor that stimulated the growth, survival and migration of endothelial cells, and promotes the development and tumour angiogenesis [24]. Ovarian tumors study showed patients with high cytoplasmic FGF2 were associated with reduced tumor aggressiveness and increased survival rates compared with patients with low expression of FGF2 [25]. Li et al. found that FGF-Trap effectively suppressed FGF-2 induced proliferation and migration of human umbilical vein endothelial cells, and FGF-Trap potently inhibited tumour growth and angiogenesis in Caki-1 and A549 xenograft models in vivo [26]. Xue et al. found that c-Myc-mediated repression of miR-15-16 in hypoxia was induced by increased HIF-2α and promoted tumor angiogenesis and metastasis by up-regulating FGF2 [27]. These findings indicated that targeting the FGF2 expression may provide a potential molecular target for the cancer therapy.

In this study, we found that miR-203 could inhibit cell proliferation, migration and invasion of renal cancer cells, which suggested that miR-203 could play a key role in the regulation of cell growth and metastasis of renal cancer cells. However, the underlying mechanisms were still unclear. In the study, we showed that FGF2 was a direct target of miR-203 and restoration of miR-203 could down-regulate the expression of FGF2 in 786-O cells. The restoration of FGF2 markedly attenuated tumor suppressive effects of miR-203 on renal cancer cells. Furthermore, FGF2 levels were increased and negatively correlated with miR-203 levels in ccRCC tissues. Taken together, these data suggested that miR-203 impacted on renal cancer cells partially by inactivation of FGF2.

## Conclusions

In conclusion, our results provided evidence that miR-203 was dramatically down-regulated in renal cancer and the decreased expression of miR-203 expression was associated with more aggressive tumor phenotype and poorer overall survival in ccRCC patients. Ectopic expression of miR-203 could inhibit cell proliferation, migration and invasion capacity of renal cancer cells by targeting FGF2. These results suggested that miR-203 could be a useful prognostic marker and a potential therapeutic target in human renal cancer.
